# A Method for Evaluating the Performance of Main Bearings of TBM Based on Entropy Weight–Grey Correlation Degree

**DOI:** 10.3390/s25154715

**Published:** 2025-07-31

**Authors:** Zhihong Sun, Yuanke Wu, Hao Xiao, Panpan Hu, Zhenyong Weng, Shunhai Xu, Wei Sun

**Affiliations:** 1 School of Mechanical Engineering, Dalian University of Technology, Dalian 116024, China; goszh@sohu.com (Z.S.); sunwei@dlut.edu.cn (W.S.); 2 China Railway Engineering Equipment Group Co., Ltd., Zhengzhou 450016, China; xh032016@gmail.com (H.X.); hupanpan@crectbm.com (P.H.); zhenyongweng@163.com (Z.W.); xushunhai@crectbm.com (S.X.)

**Keywords:** TBM, main bearing, experimental test, performance evaluation

## Abstract

The main bearing of a tunnel boring machine (TBM) is a critical component of the main driving system that enables continuous excavation, and its performance is crucial for ensuring the safe operation of the TBM. Currently, there are few testing technologies for TBM main bearings, and a comprehensive testing and evaluation system has yet to be established. This study presents an experimental investigation using a self-developed, full-scale TBM main bearing test bench. Based on a representative load spectrum, both operational condition tests and life cycle tests are conducted alternately, during which the signals of the main bearing are collected. The observed vibration signals are weak, with significant vibration attenuation occurring in the large structural components. Compared with the test bearing, which reaches a vibration amplitude of 10 g in scale tests, the difference is several orders of magnitude smaller. To effectively utilize the selected evaluation indicators, the entropy weight method is employed to assign weights to the indicators, and a comprehensive analysis is conducted using grey relational analysis. This strategy results in the development of a comprehensive evaluation method based on entropy weighting and grey relational analysis. The main bearing performance is evaluated under various working conditions and the same working conditions in different time periods. The results show that the greater the bearing load, the lower the comprehensive evaluation coefficient of bearing performance. A multistage evaluation method is adopted to evaluate the performance and condition of the main bearing across multiple working scenarios. With the increase of the test duration, the bearing performance exhibits gradual degradation, aligning with the expected outcomes. The findings demonstrate that the proposed performance evaluation method can effectively and accurately evaluate the performance of TBM main bearings, providing theoretical and technical support for the safe operation of TBMs.

## 1. Introduction

**Figure 1:**
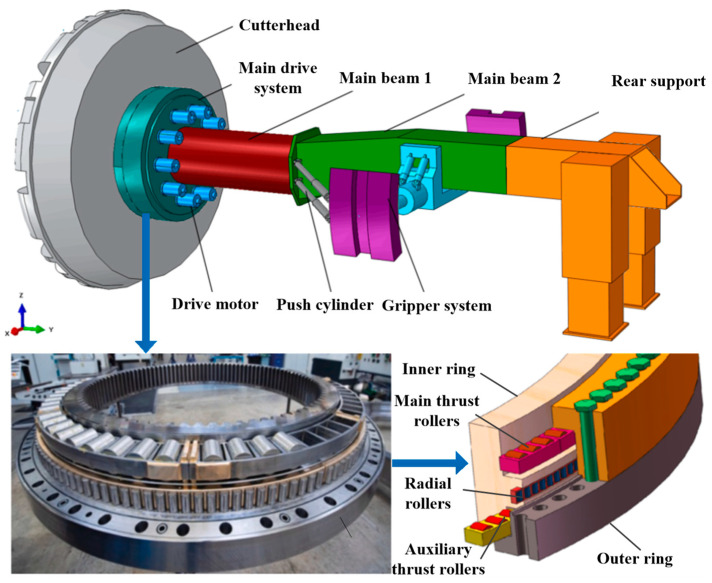
The structural diagram of a TBM and its main bearings [4,5].

A tunnel boring machine (TBM) is essential for underground space construction, and its technological advancement has become an important indicator of a country’s high-end equipment manufacturing capabilities [1,2,3]. Figure 1 illustrates the TBM structure, including the cutterhead, main drive system, traction motor, main beam 1, push cylinder, gripper system, main beam 2, and rear support [4,5]. The main bearing is a key component of the main drive system, allowing the performance of continuous tunneling by bearing and transmitting loads [6,7,8]. Most TBM main bearings are slewing bearings, usually consisting of three rows of cylindrical roller bearings. The main bearing comprises an inner ring, an outer ring, main thrust rollers, auxiliary thrust rollers, and radial rollers. The thrust rollers are mainly used to transmit the axial load and capsizing moment, while the radial rollers withstand the radial load and the gravity of the main bearing. Additionally, the auxiliary thrust rollers help resist the overturning moment, ensuring stable operation [7,8,9,10,11].

TBM main bearings are low-speed, heavy-duty rolling bearings. Their diameters can reach several meters (sometimes exceeding ten meters), with rotational speeds below 10 r/min and axial loads up to tens of thousands of kilonewtons. These bearings have very long manufacturing and replacement cycles and require advanced technology, special equipment, and strict environmental conditions. Typically, the service life of the main bearing system is equivalent to that of the TBM itself. Ensuring the safety and reliability of the main bearings is therefore critical to the safe operation of the TBM [12,13,14].

TBMs operate underground, making the underlying geological environment and working conditions critical to their efficient operation. The unique structure and working conditions pose significant challenges in evaluating bearing performance. The health of the main bearing is vital to TBM efficiency, as failure modes such as wear, spalling, cracking, and rusting can cause irreversible damage to TBM components [15,16,17]. Zhang et al. [4] investigated the spatiotemporal damage evolution laws of the main bearing using finite element analysis. Their findings indicated that the damage risk of the raceway is greater than that of the rollers, and that of the main push raceway is greater than that of the other two raceways. Zhang et al. [12] studied the residual strength of roller–raceway contact using a stress–strength interference model that accounted for nonlinear strength degradation and material dispersion, and calculated the reliability of the main bearing structure. Fu et al. [18] proposed a two-stream convolutional neural network with multi-channel detrending input (TSCNN-MCDI) to analyze three-axis vibration signals from an accelerometer inside the roller, enabling the detection of faults in either the roller or the entire raceway. Peng [19] developed a comprehensive tool load model and a main bearing load distribution model for composite rock strata, and investigated the impact of the condition of the strata on the distribution of external and internal loads on the main bearing. Wang et al. [20] proposed a hierarchical finite element modeling method in which constant contact pressure is replaced with rolling contact to examine the problems of low hardness and easy fatigue spalling in the soft zone of TBM main bearings.

Numerous studies have focused on developing models for the accurate analysis of the dynamic characteristics of bearings. For instance, He et al. [21] studied the load-bearing capacity of single-row four-point contact ball slewing bearings, and optimized parameters such as the number of balls and their diameter. Zhang et al. [22] determined the load distribution of slewing bearings in wind turbine units based on rigid and flexible rings, and provided suggestions for their structural parameters. Han et al. [23] conducted a finite element analysis of the TBM main bearing under radial loads using the ANSYS software, and analyzed its internal contact stresses and load distribution. Zong et al. [24] developed a dynamic model using the Abaqus software to simulate raceway defects in slewing bearings, and determined the vibration responses of bearings caused by defects of varying sizes. Huo et al. [2] developed a novel test bench for hard rock TBM main systems and established a multi-degree-of-freedom coupled dynamic model, which they validated through experiments under typical working conditions. Cao et al. [25] developed a dynamic model of the TBM main bearing that incorporated local defects; they investigated the effects of uneven loads on the vibration of the main bearing and revealed the vibration mechanisms associated with these defects. Zhang et al. [26] characterized the time-varying displacement excitation of rolling elements to describe the changes in contact clearance between the rolling elements and their inner and outer rings. They also proposed the time-varying impact force excitation function for defective rolling elements, and analyzed how the rotational speed and defect size influence the fault vibration characteristics of the rolling elements.

Fault diagnosis and performance evaluation primarily depend on monitoring the operating status of the bearing during use to predict its reliability or remaining service life [27,28,29,30,31,32,33]. Bastami et al. [34] utilized specific vibration patterns generated by defective rolling bearings as diagnostic tools for predictive maintenance. They simulated and analyzed the vibration signals of the rolling bearings using physical models to represent natural defects and established the relationships between the defect size and vibration characteristics. Minhas et al. [35] proposed a bearing fault diagnosis method combining complementary set empirical mode decomposition (EMD) and weighted multi-scale entropy to address the problem of strong noise in vibration signals. They validated the effectiveness of their method using actual acoustic signal data. Pancaldi et al. [36] proposed a fault detection method for rolling bearings based on the statistical definition of cyclic stationary states, which demonstrated high robustness against mechanical noise. Ma et al. [37] proposed a state detection method for DBN tailored to non-stationary signals generated by bearings operating under variable-speed conditions. They optimized the method using the sparrow search algorithm and used fault features extracted via the Fourier transform for the classification, identification, and diagnosis of bearing faults. Zareapoor et al. [38] addressed the problem of class imbalance in industrial signal data by proposing a generative adversarial network for minority class oversampling. This method generates new minority class samples by combining the majority class distribution and employs a discriminator for fault diagnosis. Zhao et al. [39] proposed a novel adaptive decomposition algorithm based on CEEMDAN and fractal dimension analysis to overcome issues such as redundancy and mode confusion in traditional EMD-based algorithms. Chen et al. [40] proposed a residual deep subdomain adaptive network for intelligent bearing fault diagnosis across multiple domains, with the key advantage that only one transfer task is required regardless of changes in operating conditions.

Although the aforementioned studies achieved promising results, most were based on small-scale experiments. Only a few full-scale testing platforms are capable of evaluating the main bearings of TBMs. Notable facilities with such advanced testing abilities include SKF’s Sven Wingquist Test Center, Schaeffler’s FAG bearing ASTRAIOS Test Platform, Liebherr’s Large Bearing Test Platform, Wafangdian Bearing Group Corp., Ltd.’s 3.6 m large fan bearing test bench, and Luoyang LYC Bearing Corp., Ltd.’s comprehensive performance test bench for slewing bearings. Due to the large structures of full-scale test benches and the limited number of test samples, few experimental studies have been conducted on the performance evaluation of TBM main bearings. Research in this area remains in its early stages and lacks the capability to accurately evaluate and predict bearing health, or to provide timely and reliable information for intelligent operation and maintenance.

In summary, a dedicated testing technology for TBM main bearings does not yet exist. Therefore, in this work, a full-scale (1:1) TBM main bearing test bench was established to conduct an experimental study based on the test load spectrum. A performance evaluation method for TBM bearings, combining the entropy weight and grey relational degree, is proposed. This method relies on the collection of main bearing signals during testing and the selection of evaluation indices that reflect the operational state of the bearing. The results provide technical support for the safe operation of TBM main bearings.

## 2. Main Bearing Test

### 2.1. Test Bench Introduction

**Figure 2:**
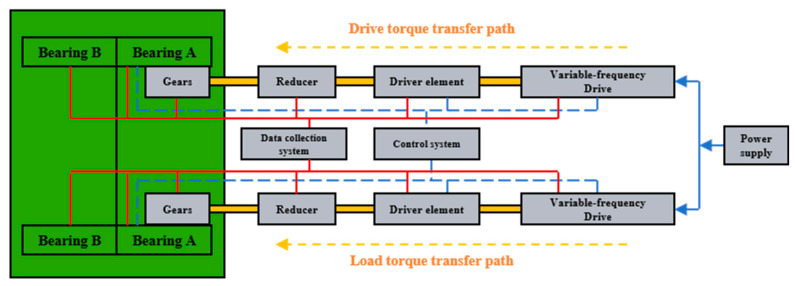
The schematic of the test bench loading system.

**Figure 3:**
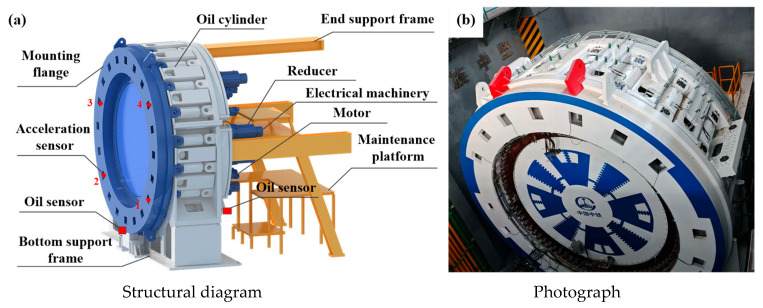
The structure of the test bench.

A systematic study was conducted by designing a full-scale test bench for the main bearing based on the main drive structure of the TBM, as shown in Figure 1. The test bench accommodates replaceable bearings with diameters ranging from 3 to 6 m. The loading principle is illustrated in Figure 2. Two bearings are arranged back-to-back to eliminate the influence of the bearing rotation pair on the applied load. The axial force, radial force, and overturning moment are applied via evenly distributed hydraulic cylinders, each capable of independent loading to simulate various load conditions. The inner gear ring of the main bearing is driven by driving motors, while the load torque is provided by a separate load motor. The load conditions of the test bearing are consistent with real working conditions. The test bench is equipped with a comprehensive control, data acquisition, and monitoring system capable of tracking various signals, such as the bearing load, torque, speed, temperature, and vibration. Four vibration acceleration sensors are installed on the two test bearings in both the axial and radial directions, and an online oil sensor is positioned at the front end of the filter valve. The structure of the test bench is shown in Figure 3. In addition to the test system, the test bench includes personnel channels and maintenance platforms to ensure its safe and reliable operation.

### 2.2. Testing Program

**Figure 4:**
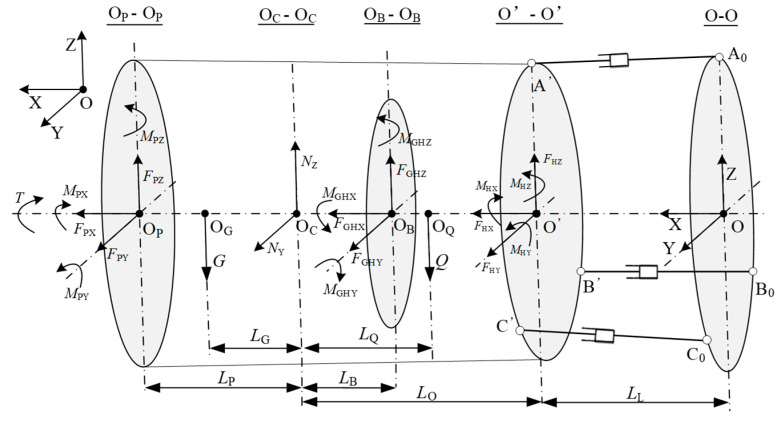
The simplified mechanical model of the main drive structure [41].

In a previous study [41], a mechanical model of the TBM main drive was developed and the relationship between the main bearing load and the cylinder attitude was identified, as illustrated in Figure 4. This model enabled the determination of the main bearing load for the TBM. A typical geological section was selected to analyze the load spectrum experienced by the main bearing, resulting in the identification of eight load spectrum conditions, as summarized in Table 1 [42].

In this study, tests were conducted according to these load conditions. An empty running test was performed between different working conditions to eliminate their mutual influence. Moreover, an accelerated life cycle test with a duration of 20 days was conducted after each working condition. The life cycle test and working condition test were alternately conducted to simulate the full bearing life cycle. Each working condition test (including the empty running test) had a duration of 5 min, during which the signals were collected.

## 3. Data Analysis

### 3.1. Characteristic Parameters

Time-domain statistical parameters can be derived from the system’s time-domain signals and are commonly used to evaluate its operating status. These parameters are generally classified into two categories: dimensional parameters (i.e., peak value, root mean square (RMS), and variance) and dimensionless parameters (i.e., peak factor, waveform factor, pulse factor, margin factor, and kurtosis). In this study, the characteristic parameters selected for comprehensive evaluation included the peak factor, RMS, margin factor, kurtosis, lubricating oil temperature, and oil kinematic viscosity. These indicators were used to analyze and evaluate the state of the bearing throughout the test process.

### 3.2. Analysis of Vibration Signal Results

**Figure 5:**
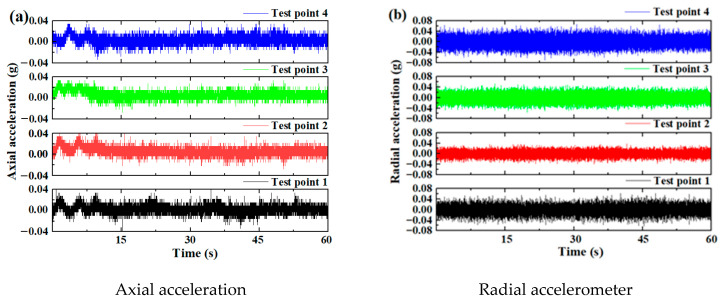
The vibration time-domain signal of Test Bearing A.

**Figure 6:**
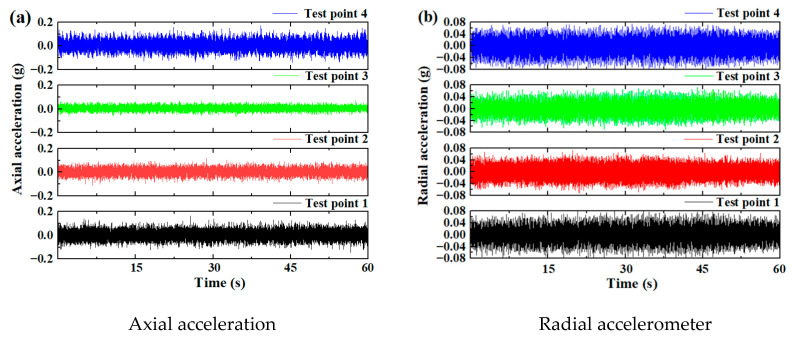
The vibration time-domain signal of Test Bearing B.

Figure 5 and Figure 6 show the vibration signals of two test bearings under an axial force of 13,846 kN, a radial force of 6954 kN, and an overturning moment of 5352 kN·m (i.e., Working Condition 1). For Test Bearing A, the amplitude of the four axial vibration accelerations was 0.04 g, while the four radial vibration acceleration amplitudes were approximately 0.08 g. In contrast, the four axial vibration acceleration amplitudes of Test Bearing B reached 0.2 g, with radial vibration acceleration amplitudes equal to 0.08 g. The radial vibration amplitudes of the two bearings were comparable, whereas the axial vibration amplitudes differed by an order of magnitude. This discrepancy may have been due to the vibration attenuation caused by the different positions of the vibration acceleration sensors. Overall, the vibration levels of the bearings were significantly lower than those of the entire TBM. During the testing of a shield machine in a previous study, the bearing vibration was more than ten times greater than that of the entire shield machine [43]. The vibration amplitude observed in this test was also several orders of magnitude lower than the 10 g amplitude recorded in scaled tests of the main TBM bearing in a previous study [25]. Because only the bearings were tested here, the measured vibration was low. This is primarily due to the significant attenuation during vibration transmission through large structural components, which poses a challenge for accurately testing the main bearings of the shield machine.

Figure 5 and Figure 6 also illustrate that vibration signals alone are insufficient to effectively reflect the operating state of the bearings. Therefore, various data fusion approaches were adopted for bearing performance evaluation. The effective value, kurtosis, peak factor, and margin factor of the vibration signals, as well as the lubricating oil temperature and oil kinematic viscosity, of Test Bearings A and B were selected as performance evaluation indices. These indicators were calculated based on data collected by the test bench.

## 4. Performance Evaluation Method Based on Entropy Weight

The entropy weight method was employed to calculate the weight of each evaluation index. The performance of the TBM main bearing was then evaluated via a combination of the entropy weight method and grey relational analysis. Grey relational analysis is particularly suitable for this application due to its ability to effectively excavate intrinsic patterns in test data along with its low data requirements, ease of calculation, and straightforward implementation [44,45,46]. The entropy weight method eliminates the influence of subjective factors on weighting, allowing for an objective assessment of indicator importance. This approach has demonstrated high accuracy in practical applications [47,48,49,50]. As an objective weighting technique, it employs information entropy to calculate the weight coefficient of each indicator based on the degree of variation between the indicators. The calculation process is summarized as follows.

The proportion of the index value (*P*_ij_) of the *j*-th item in scheme *i* is computed as follows.

(1) $$P_{ij}=x_{ij}(j)/\sum_{i=1}^{n}x_{i}(j)$$

The entropy ($E_{j}$) of the *j*-th index is calculated as

(2) $$E_{j}=-k/\sum_{i=1}^{n}P_{ij}\ln P_{ij}$$

where k=1/lnn.

The difference coefficient ($g_{i}$) of the *j*-th index is computed as follows.

(3) $$g_{j}=1-E_{j}$$

The weight ($\omega_{i}$) of the *j*-th indicator is calculated as follows.

(4) $$\omega_{j}=g_{j}/\sum_{j=1}^{m}g_{j}$$

The final entropy weight vector is then obtained:

(5) $$W=(\omega_{1},\omega_{2},\ldots ,\omega_{m})$$

### 4.1. Calculation of Grey Relational Coefficient

Correlations often exist among the indicators used in monitoring methods. The degree of association between each indicator can be determined using the grey relational coefficient. A set of reference indicators is selected as the comparison sequence, which is compared against the decision series to assess the degree of correlation. The calculation process is summarized as follows.

The reference sequence (*x*_0_) and decision sequence (*x_i_*) are set as follows.

$$x_{0}=\left\{x_{0}(j)|j=1,2,\ldots ,m\right\}=\{x_{0}\left(1\right),x_{0}\left(2\right),\ldots ,x_{0}\left(m\right)\}$$

$$x_{i}=\left\{x_{i}(j)|j=1,2,\ldots ,m;i=1,2,\ldots ,n\right\}=\{x_{i}\left(1\right),x_{i}\left(2\right),\ldots ,x_{i}\left(m\right)\}$$

The aforementioned sequence is normalized by adopting the range normalization treatment:

(6) $$x=\frac{x_{j}-min(x_{j})}{max(x_{j})-min(x_{j})}$$

The correlation coefficient is given as follows:

(7) $$\xi_{i}\left(j\right)=\frac{\frac{min}{n}\frac{min}{m}\left|x_{0}\left(t\right)-x_{s}(t)\right|+\rho \frac{max}{n}\frac{max}{m}\left|x_{0}\left(t\right)-x_{s}(t)\right|}{\left|x_{0}\left(t\right)-x_{i}(t)\right|+\rho \frac{max}{n}\frac{max}{m}\left|x_{0}\left(t\right)-x_{s}(t)\right|}$$

where i=1,2,…,n;j=1,2,…,m,$\frac{min}{n}\frac{min}{m}\left|x_{0}\left(t\right)-x_{s}(t)\right|$, and $\frac{max}{n}\frac{max}{m}\left|x_{0}\left(t\right)-x_{s}(t)\right|$ are, respectively, the two-stage minimum and maximum differences, and *p* is a parameter usually set in the range of 0.5–0.6.

### 4.2. Main Bearing Performance Evaluation

Based on the previously calculated weight vector and grey relational coefficient matrix, a comprehensive evaluation is performed to determine the relative correlation degree, as given by the following equation.

(8) $$R=W\xi^{T}$$

To assess bearing performance, test data from various working conditions are used for a first-level comprehensive evaluation. This yields a decision matrix corresponding to each working condition, representing its classification type.

(9) $$R_{M}=\left[\begin{array}{c} R_{1}=W_{1}\xi_{1}\\ \begin{array}{c} R_{2}=W_{2}\xi_{2}\\ \begin{array}{c} \ldots \\ R_{k}=W_{k}\xi_{k} \end{array} \end{array} \end{array}\right]$$

The second-level comprehensive evaluation is then conducted based on the decision matrix obtained in the first level and following the previously described calculation steps. Additionally, the weight vector for each working condition is computed using Equations (1)–(5). These weights are applied to obtain the final second-level comprehensive evaluation result $ER=\left[ER_{1},ER_{2},\ldots ,ER_{n}\right]$:

(10) $$ER=W_{M}\cdot R_{M}^{T}$$

where $ER_{n}$ represents the comprehensive evaluation coefficient of the relative reference under the *n*-th working condition.

## 5. Performance Evaluation of the Main Bearing

### 5.1. Comprehensive Bearing Performance Evaluation Under Different Loading Conditions

The effective value, kurtosis, peak value, and margin of the vibration signals, as well as the lubricating oil temperature and oil kinematic viscosity, of Test Bearings A and B were selected as performance evaluation indices. Tests were conducted under the eight load conditions presented in Table 1, and the rotational speed was set to 0.5 r/min, corresponding to the typical operating speed of the TBM. The performance indices for both bearings were obtained and are presented in Table 2.

After applying normalization using Equation (6), the normalized main bearing evaluation indices under the eight loading conditions at a speed of 0.5 r/min were calculated. The results are reported in Table 3.

The entropy weights were calculated via Equations (1)–(5), with the results as follows:

W = [0.095, 0.038, 0.047, 0.048, 0.100, 0.084, 0.042, 0.052, 0.138, 0.098, 0.150, 0.108].

**Figure 7:**
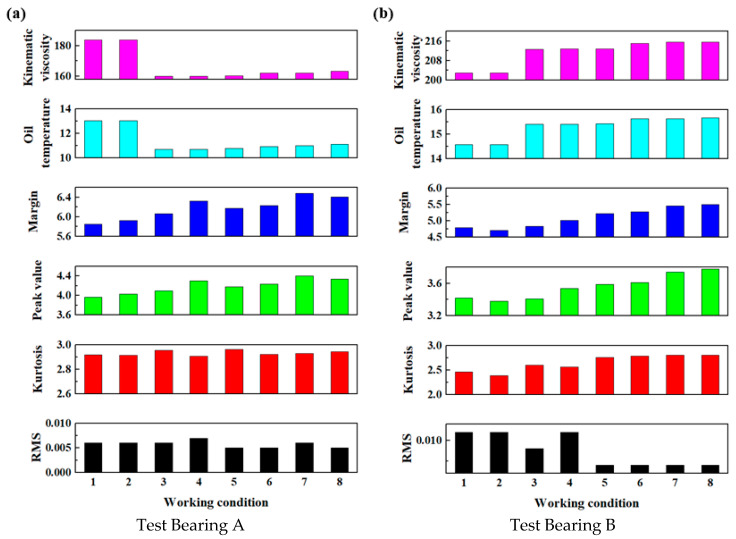
The correlation coefficients of the performance indices of the main bearing under the eight loading conditions at a speed of 0.5 r/min.

The correlation coefficients of the performance indices of the main bearing under the eight loading conditions at a speed of 0.5 r/min were calculated using Equation (7). The results are visualized in Figure 7.

The evaluation coefficients under these working conditions were then calculated using Equation (8), with the following results:

*R*_1_ = *W*_1_*ξ*_1_ = [1, 0.9071, 0.5053, 0.5186, 0.4224, 0.4282, 0.4027, 0.3978],

Similarly, the evaluation coefficients under the eight working conditions at speeds of 1, 1.5, and 2 r/min were calculated, with the following results:

*R*_2_ = *W*_2_*ξ*_2_ = [1, 0.6524, 0.6053, 0.5192, 0.5176, 0.4747, 0.4301, 0.3803],

*R*_3_ = *W*_3_*ξ*_3_ = [1, 0.5946, 0.5903, 0.5111, 0.5556, 0.5284, 0.4876, 0.4158],

*R*_4_ = *W*_4_*ξ*_4_ = [1, 0.8387, 0.6940, 0.6361, 0.6601, 0.5414, 0.6680, 0.4998].

**Figure 8:**
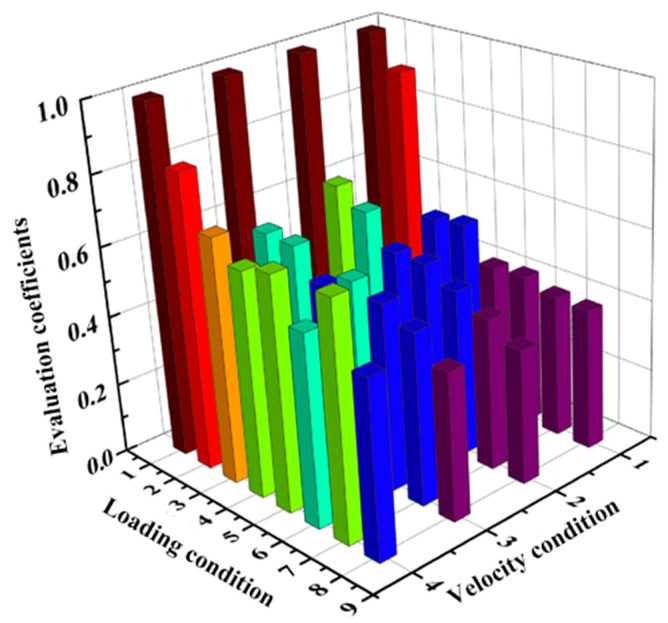
The evaluation coefficients under different working conditions.

The evaluation coefficients under 32 different working conditions were obtained, and the results are shown in Figure 8. It can be seen from the Figure 8 that the variation trends of the evaluation coefficients of the bearing under different working conditions.

The entropy weight values under different speeds were computed via Equation (9), as follows:

$$W_{M}=\left[\begin{array}{cc} 0.4033 & \begin{array}{ccc} 0.2611 & 0.2045 & 0.1311 \end{array} \end{array}\right]$$

$$R_{M}=\left[\begin{array}{cccc} 1 & 1 & 1 & 1\\ 0.9071 & 0.6524 & 0.5946 & 0.8387\\ 0.5053 & 0.6053 & 0.5903 & 0.6940\\ 0.5186 & 0.5192 & 0.5111 & 0.6361\\ 0.4224 & 0.5176 & 0.5556 & 0.6601\\ 0.4282 & 0.4747 & 0.5284 & 0.5414\\ 0.4027 & 0.4301 & 0.4876 & 0.6680\\ 0.3978 & 0.3803 & 0.4158 & 0.4998 \end{array}\right]$$

*ER* = *W_M_R_M_^T^* = [1, 0.7677, 0.5735, 0.5326, 0.5057, 0.4757, 0.4620, 0.4103].

**Figure 9:**
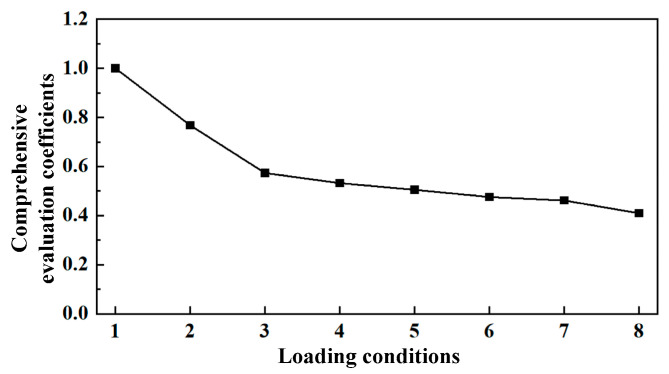
The comprehensive evaluation coefficients under different loading conditions.

Figure 9 presents the comprehensive evaluation coefficients under different loading conditions. Using the first working condition as a reference, it can be seen that the closer the bearing performance was to this condition, the closer the comprehensive evaluation coefficient was to the theoretical value of one. Additionally, as the bearing load increased, the comprehensive evaluation coefficient decreased. Therefore, it can be concluded that the entropy weight–grey relational evaluation method can accurately evaluate the bearing performance.

### 5.2. Bearing Performance Evaluation Under Cyclic Working Conditions

To study the evolution of the bearing performance under the same working conditions, a set of prior working condition tests was used as a reference. Life cycle tests were then conducted to analyze the subsequent test data across multiple repeated working cycles. The corresponding evaluation coefficient (*R*) was calculated using the entropy weight–grey relational degree evaluation method. To minimize discrepancies caused by varying test references, a relative evaluation coefficient was used to assess the life cycle tests. The differences were calculated based on the obtained evaluation coefficients $R_{1},R_{2},\ldots ,R_{n}$, and the relative error evaluation coefficient $∆R_{i}(i=1,2,\ldots ,n-1)$ was computed as follows.

(11) $$∆R_{i}=R_{i+1}-R_{i}$$

The relative evaluation coefficient was then calculated as follows.

(12) $$CR_{i}=1-∆R_{i}$$

Five life cycle tests were selected for analysis, with repeated test conditions of a speed of 1 r/min and a loading force of 37,500 kN. The resulting evaluation indices are reported in Table 4.

**Figure 10:**
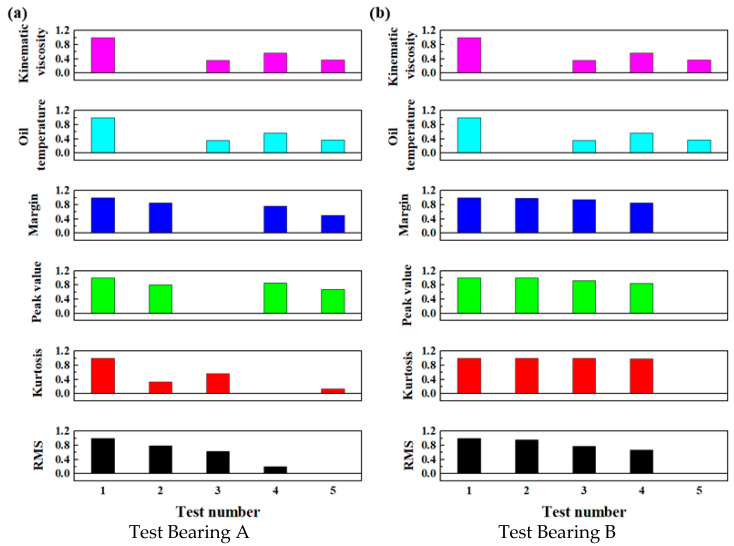
The normalized evaluation indices of the main bearings under a loading force of 32,500 kN and a speed of 1 r/min.

After applying normalization using Equation (6), the main bearing evaluation indices under a normalized loading force of 37,500 kN and speed of 1 r/min were calculated. The results are exhibited in Figure 10.

Using the data from the first test as the reference and the remaining test data as the calculation input, the entropy weights were calculated according to Equations (1)–(5), as follows:

W = [0.1353, 0.1697, 0.0921, 0.0994, 0.026, 0.772, 0.115, 0.112, 0.1259, 0.1259, 0.1259, 0.1259].

**Figure 11:**
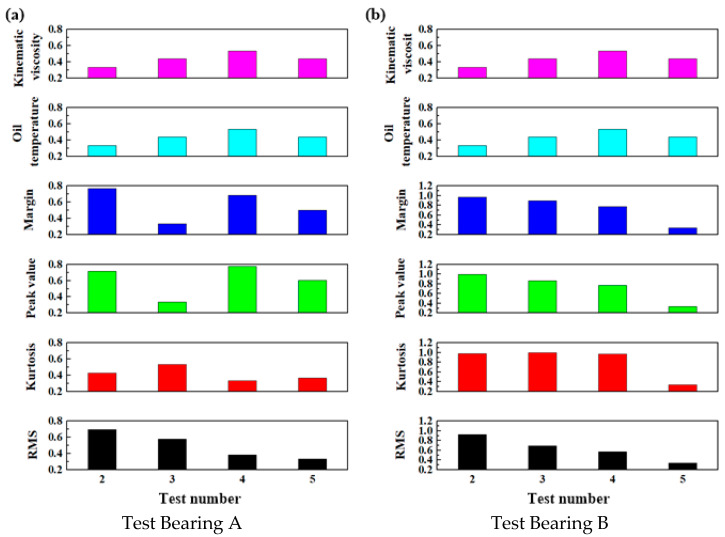
The correlation coefficients of the main bearings under a loading force of 32,500 kN and a speed of 1 r/min.

The correlation coefficients of the main bearing under a loading force of 32,500 kN and a speed of 1 r/min were then calculated using Equation (3). The results are presented in Figure 11.

The relative errors and relative evaluation coefficients of the two bearings were respectively calculated using Equations (10) and (11), with the following results.

Relative error:

△*R*_A_ = [0.0462, 0.1220, 0.1578]

△*R*_B_ = [0.0104, 0.0436, 0.0606]

Relative evaluation coefficient:

△*CR*_B_ = [0.9538, 0.8780, 0.8422]

△*CR*_B_ = [0.98, 0.95, 0.87]

**Figure 12:**
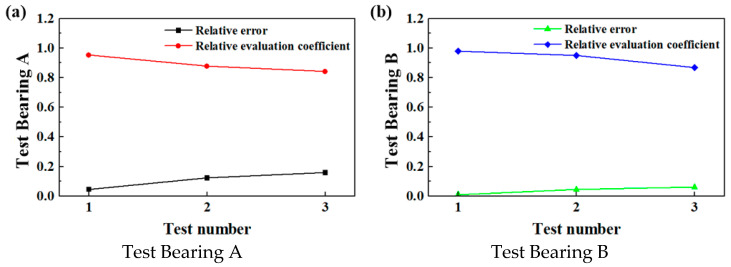
The relative evaluation coefficients under different test cycles.

Figure 12 shows the relative evaluation coefficients under different test cycles. As the test duration increased, the relative evaluation coefficients of the bearings gradually decreased, while the relative evaluation errors gradually increased. Each group of working conditions underwent a 20-day life cycle test. The observed degradation in bearing performance over time is consistent with the downward trend of the relative evaluation coefficient.

## 6. Conclusions

In this study, tests were conducted on a self-designed, full-scale (1:1) test bench for the main bearing of a TBM to evaluate its performance. The test conditions were chosen according to a typical load spectrum, and a 20-day life cycle test was conducted after each set of working conditions. The data collected during the test process were used to evaluate the bearing performance using the entropy weight–grey relational method. The conclusions of this work are as follows.

(1) During the shield machine main bearing test, the vibration signal amplitude was low, with significant attenuation caused by large structural components. This resulted in vibration amplitudes several orders of magnitude lower than the 10 g amplitude observed in scale tests of the main bearing.
(2) To effectively utilize the selected evaluation indicators, the entropy weight method was employed to assign their weights. Furthermore, a comprehensive analysis combining the entropy weight and grey relational methods was developed, resulting in a unified evaluation method that determines the comprehensive evaluation coefficient.
(3) Using the first working condition as a reference, the bearing performance closer to this condition corresponded to a comprehensive evaluation coefficient nearer to the theoretical value of one. As the bearing load increased, the comprehensive evaluation coefficient decreased, indicating a decline in bearing performance.
(4) A multistage comprehensive evaluation method was applied to assess the performance and condition of the main bearing under multiple working conditions. Over time, with the increase of the test duration, the bearing performance gradually degraded.

## Figures and Tables

**Table 1:** The composite load spectrum of the entire section [42].

Working Condition	*F_a_*/kN	*F_r_*/kN	*M_r_*/kN·m	Percentage	Frequency	Cumulative Proportion	Cumulative Frequencies
1	13,845.978	6953.7756	5352.6227	0.275	275,000	0.275	275,000
2	15,473.515	7026.2071	6826.2635	0.15	150,000	0.425	425,000
3	16,361.415	7065.7219	7630.2052	0.15	150,000	0.575	575,000
4	17,249.315	7105.2367	8434.1469	0.15	150,000	0.725	725,000
5	18,199.745	7147.5343	9294.7057	0.125	125,000	0.85	850,000
6	19,314.514	7197.1457	10,304.065	0.1	100,000	0.95	950,000
7	20,877.751	7266.7155	11,719.485	0.049998	49,998	0.999998	999,998
8	27,314.803	7553.1881	17,547.862	2.00 × 10^−6^	2	1	1,000,000

**Table 2:** The performance indices of the main bearing under eight loading conditions and a rotational speed of 0.5 r/min.

Conditions		1	2	3	4	5	6	7	8
Performance Index									
Bearing A	RMS	0.006	0.006	0.006	0.007	0.005	0.005	0.006	0.005
	Kurtosis	2.920	2.916	2.958	2.908	2.965	2.924	2.930	2.945
	Peak value	3.962	4.030	4.099	4.302	4.181	4.237	4.404	4.340
	Margin	5.846	5.925	6.060	6.324	6.174	6.231	6.485	6.406
	Oil temperature	13.038	13.038	10.711	10.711	10.767	10.923	10.978	11.112
	Kinematic viscosity	183.945	183.945	159.960	159.960	160.277	161.968	161.992	163.203
Bearing B	RMS	0.011	0.011	0.009	0.011	0.007	0.007	0.007	0.007
	Kurtosis	2.460	2.392	2.601	2.561	2.757	2.791	2.803	2.808
	Peak value	3.420	3.379	3.406	3.536	3.585	3.608	3.737	3.775
	Margin	4.785	4.699	4.838	5.017	5.218	5.273	5.464	5.501
	Oil temperature	14.566	14.566	15.413	15.413	15.436	15.625	15.625	15.672
	Kinematic viscosity	202.976	202.976	212.60	212.704	212.704	215.030	215.509	215.509

**Table 3:** The normalized performance indices of the main bearing under eight loading conditions and a rotational speed of 0.5 r/min.

Condition		1	2	3	4	5	6	7	8
Performance Index									
Bearing A	RMS	1.000	0.731	0.237	0.158	0.000	0.038	0.166	0.082
	Kurtosis	1.000	0.914	0.156	0.733	0.000	0.913	0.779	0.451
	Peak value	1.000	0.847	0.691	0.232	0.505	0.379	0.000	0.145
	Margin	1.000	0.877	0.665	0.253	0.488	0.399	0.000	0.124
	Oil temperature	1.000	1.000	0.000	0.000	0.024	0.091	0.115	0.172
	Kinematic viscosity	1.000	1.000	0.000	0.000	0.013	0.084	0.085	0.135
Bearing B	RMS	1.000	0.970	0.440	0.969	0.017	0.061	0.000	0.026
	Kurtosis	1.000	0.804	0.596	0.709	0.147	0.049	0.014	0.000
	Peak value	1.000	0.884	0.958	0.675	0.536	0.471	0.109	0.000
	Margin	1.000	0.880	0.926	0.676	0.395	0.318	0.052	0.000
	Oil temperature	1.000	1.000	0.234	0.234	0.213	0.042	0.042	0.000
	Kinematic viscosity	1.000	1.000	0.231	0.224	0.224	0.038	0.000	0.000

**Table 4:** The main bearing evaluation indices under a loading force of 32,500 kN and a speed of 1 r/min.

Conditions		First	Second	Third	Fourth	Fifth
Performance Index						
Bearing A	RMS	0.054	0.055	0.057	0.060	0.061
	Kurtosis	2.662	2.589	2.615	2.554	2.568
	Peak value	3.619	3.634	3.543	3.630	3.594
	Margin	5.184	5.162	5.035	5.149	5.109
	Oil temperature	19.103	20.745	20.156	19.824	20.144
	Kinematic viscosity	260.994	262.636	262.047	261.715	262.035
Bearing A	RMS	0.0010	0.0010	0.001033	0.001038	0.0010
	Kurtosis	3.956	4.075	3.940	4.117	5.454
	Peak value	4.68	4.66	4.99	5.27	8.56
	Margin	6.986	7.086	7.314	7.821	12.695
	Oil temperature	26.259	27.901	27.312	26.980	27.300
	Kinematic viscosity	306.927	308.569	307.980	307.648	307.968

## Data Availability

Due to industry confidentiality, no data is available.
